# A novel anti-loosening bolt looseness diagnosis of bolt connections using a vision-based technique

**DOI:** 10.1038/s41598-024-62560-8

**Published:** 2024-05-20

**Authors:** Jun Luo, Kaili Li, ChengQian Xie, Zhitao Yan, Feng Li, Xiaogang Jia, Yuanlai Wang

**Affiliations:** 1 School of Civil Engineering and Architecture, Chongqing University of Science and Technology, No. 20, East University Town Road, Shapingba District, Chongqing, 401331 China; 2 Chongqing Urban Investment Infrastructure Construction Co., Ltd, Chongqing, China

**Keywords:** Bolt connections, Anti-loosening bolts, Looseness detection, Vision-based technique, Hexagonal border reconstruction, Civil engineering, Energy infrastructure

## Abstract

Bolt looseness detection is a common problem in engineering. Most vision-based detection techniques focus on diagnosing ordinary bolt looseness, i.e., the methods used for diagnosis are based only on the sidelines of nuts. These methods cannot be used for anti-loosening bolt looseness diagnosis because of the simultaneous rotation of screws and nuts. Therefore, a novel anti-loosening bolt looseness diagnosis method based on a vision-based technique is proposed in this paper. First, a regular hexagonal cap was installed on the screw, which can be used as a reference for the nut. Then, to automatically distinguish the hexagonal borders of the screw cap and nut, a new hexagonal border reconstruction algorithm is proposed. Furthermore, the relative rotation angles of the screw cap and nut hexagons can be determined using the sidelines of the reconstructed hexagonal borders of the screw cap and nut. Finally, a novel anti-loosening bolt looseness diagnosis method is established by using the relative rotation angle of the regular hexagonal borders of the screw cap and nut under initial status and loose status. A prototype flange node of the transmission tower was used for experimental verification. The results show that the proposed method can effectively detect the loosening angle of anti-loosening bolts.

## Introduction

Bolted connections have been widely used in engineering structures due to their advantages of easy disassembly and maintenance, strong adaptability, and flexibility and reliability. However, because of vibration, impact, and dynamic and periodic loads, the bolts in bolted connections may loosen, which will reduce the reliability of bolted connections in structures and seriously affect their safety. Therefore, the detection of bolt looseness is highly important. First, bolt looseness detection was achieved manually by observing the surface texture of the bolt, tapping the bolt with a hammer, or measuring the bolt torque using a torque wrench^1,2^. However, these methods are inefficient, carry a high risk of personnel injury and are unable to achieve real-time detection.

To establish more efficient and nonmanual detection methods, several sensors have been introduced to detect bolt looseness, and several sensor-based methods for detecting bolt looseness have been proposed, such as the guided wave method^3,4^ and the piezoelectric impedance method^5–7^. In the guided wave method, the attenuation of ultrasonic waves in the elastic bolt connection structure is monitored, which can be used to diagnose bolt loosening. In the piezoelectric impedance method, based on the electromechanical coupling characteristics of the piezoelectric material and structure, changes in the amplitude or characteristic frequency of the piezoelectric impedance spectrum can be used to identify bolt loosening. These sensor-based methods for detecting bolt looseness can improve the accuracy of bolt loosening detection and help achieve online health monitoring. However, the cost of these sensors is high, and regular maintenance is needed. Moreover, these sensors are relatively sensitive and susceptible to external interference. Therefore, sensor-based bolt looseness detection methods are difficult to deploy and apply in large engineering structures.

Currently, with the improvement of image processing technology, vision-based techniques have been introduced for bolt looseness detection because of their lower cost, greater intelligence and greater accuracy. Based on the Canny edge detection algorithm^8^ and Hough line detection algorithm^9^ in image processing technology, the edge lines of nuts can be identified. The angles between these edge lines and the horizontal coordinate axes are related to the loosening angle of the mainstream regular hexagonal bolts^10,11^, which can be used to quantify the loosening angle. The vision-based bolt loosening detection method is stable and has high detection accuracy^12^. With the rapid development of neural networks, deep learning and image processing technology have been combined to improve the efficiency of bolt location identification^13^, detect the integrity of bolts, and classify loose and nonloose bolts^14–17^. Breakthroughs in deep learning technology have greatly improved the accuracy and efficiency of bolt looseness detection methods based on vision technology, greatly increasing their application value.

**Figure 1:**
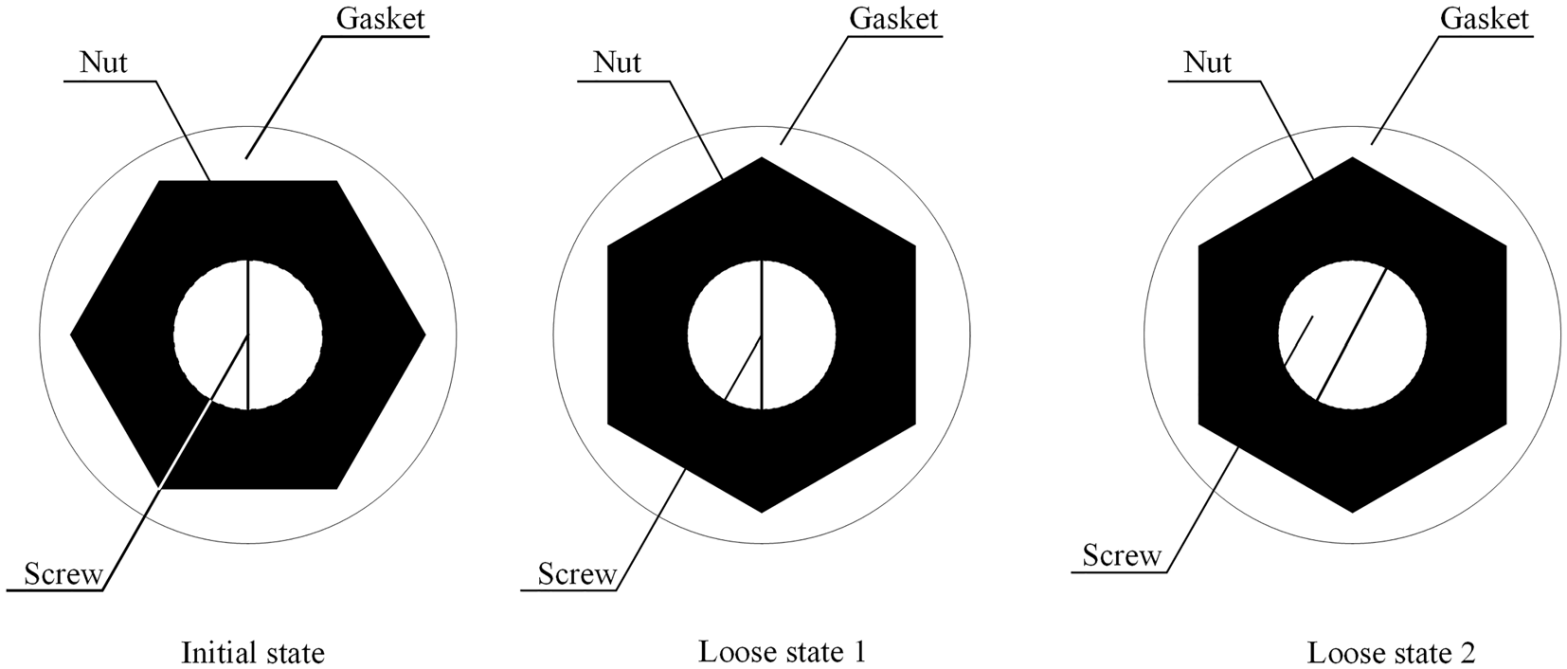
Two bolt looseness states.

However, there are still certain shortcomings in most vision-based detection methods, i.e., anti-loosening bolt looseness cannot be detected quantitatively. The reason is that the current quantitative detection methods for bolt looseness using a vision-based technique are almost exclusively based on the sideline angles of nuts. As shown in Fig. 1, under loose state 1, only the nut of the bolt rotates clockwise, and the rotation angle is 30 degrees. Therefore, using the existing quantitative detection method of bolt looseness based on vision technology, the rotation of the nut can be detected, and the conclusion that the bolt is loosened by 30 degrees can be reached. However, for anti-loosening bolts, the screw and nut may rotate together. For example, under loose state 2 in Fig. 1, the nut and screw rotate 30 degrees synchronously, and the bolt is actually not loose because there is no relative rotation between the screw and nut. Based on the existing method, the same conclusion as loose state 1 will be given, which is an incorrect conclusion. Hence, the relative rotation between the screw and nut will have a negative impact on the identification of the loosening angle.

To this end, a novel anti-loosening bolt looseness diagnosis method is proposed to quantitatively detect anti-loosening bolt looseness. First, a regular hexagonal cap was installed on the screw, which can be used as a reference for the nut. Then, to automatically distinguish the hexagonal borders of the screw cap and nut, a new hexagonal border reconstruction algorithm is proposed. Furthermore, the relative rotation angles of the screw cap and nut hexagons can be determined using the sidelines of the reconstructed hexagonal borders of the screw cap and nut. Finally, a novel anti-loosening bolt looseness diagnosis method is established by using the relative rotation angle of the regular hexagonal borders of the screw cap and nut under initial status and loose status.

The main contributions of this paper are summarized as follows:

1. A novel anti-loosening bolt looseness diagnosis method is proposed for anti-loosening bolt looseness detection. The proposed method can quantitatively detect bolt loosening with high detection accuracy and low cost.
2. A new hexagonal border reconstruction algorithm is proposed for automatically distinguishing the hexagonal borders of the screw cap and nut. The centroid of the hexagonal cap is used as the reference point, and the distances from the endpoints on the edge lines of the hexagonal borders of the screw cap and nut to the reference point are used as the clustering constraints. Then, the intra-class variance algorithm is introduced to automatically distinguish the edge lines of the hexagonal borders of the screw cap and nut, and the hexagonal borders of the screw cap and nut can be reconstructed.
3. The proposed method was verified to be effective, and the potential influencing factors were also analyzed. The results show that the proposed method in this paper can more comprehensively detect anti-loosening bolt looseness with high accuracy and good stability.

The remainder of this paper is arranged as follows. Related works on bolt loosening detection are presented in “Related Work”. The “Proposed Method” section mainly introduces the theory of the proposed novel anti-loosening bolt looseness diagnosis method. The “Experiments” section mainly verifies the feasibility of the proposed method, including method validation and analysis of the influencing factors. The “Conclusion” section contains the conclusion.

## Related work

### Bolt loosening diagnosis based on hardware sensors

Due to the inefficiency of traditional manual maintenance of bolt loosening, the detection accuracy is inadequate. In recent years, bolt looseness detection methods based on hardware sensors have attracted widespread attention from researchers. An et al.^18^ proposed a bolt loose detection method based on integrated impedance and guided waves, which utilizes piezoelectric transducers to obtain impedance and guided wave signals for bolt loose detection. Yin et al.^6^ proposed a bolt looseness detection method based on the piezoelectric active sensing method, which determines the actual contact area of bolts through ultrasonic energy transmission, thereby determining the looseness of bolts. Zhao et al.^19^ proposed a detection method for timber structural bolts based on wavelet analysis that combines the pasting of lead zirconate titanate with the time-recursive method. Zhang et al.^20^ proposed a bolt looseness detection method based on audio classification using a support vector machine, which mainly records and extracts the hammer sound produced by the varying looseness of bolt connections. Wang et al.^21^ proposed a vibroacoustic method for detecting bolt looseness. The above methods can achieve unmanned detection and sufficient detection accuracy. However, some of these sensors are relatively expensive.

### Bolt loosening diagnosis based on visual technology

Currently, with the improvement of image processing technology, vision-based techniques have been introduced for bolt looseness detection because of their lower cost, greater intelligence and greater accuracy. Ramana et al.^22^ proposed a fully automatic bolt looseness detection method based on the Viola–Jones algorithm and a support vector machine. The Viola–Jones algorithm was used to determine the bolt location, and a support vector machine was used to classify the loose bolts. Park et al.^10^ and Nguyen et al.^11^ proposed a bolt connection looseness detection technology-based Canny edge detection algorithm^8^ and Hough transform^9^, which identifies bolt loosening by comparing the sideline angles before and after bolt loosening. Kong et al.^12^ proposed a visual noncontact bolt looseness detection method by judging whether the bolt alignment process has rotated between two bolt images at different times. Cha et al.^23^ proposed a loose bolt detection method using the Hough transform and support vector machine, which can automatically calculate the damage sensitive features of bolts to train the support vector machine and construct a robust classifier to distinguish tight bolts and loose bolts. Luo et al.^24^ proposed a new bolt image correction method based on a square gasket for flange connection node bolts.

Additionally, with the rapid development of deep learning, vision-based techniques have become more intelligent and efficient. The main methods include deep learning only and the combination of deep learning and image processing technology^13–24^. Huynh et al.^13^ used convolutional neural networks (CNNs)^25^ to identify bolts and segment bolt images, and the nut edge line angles from the initial and current states were used to diagnose bolt looseness based on image processing technology. Zhang et al.^15^ used the Faster-RCNN^26,27^ deep learning algorithm for autonomous bolt looseness detection based on different screw heights, and the experimental results showed that the average accuracy of looseness detection reached 0.9503. Yang et al.^16^ comparatively tested the detection performance of three YOLO target detection algorithms^28^ for bolt loosening identification, namely, YOLO v3, YOLO v4 and YOLO v4-Tiny. Yuan et al.^29^ proposed an automatic bolt loosening identification method based on Mask-RCNN^30^. Gong et al.^31^ proposed a quantitative bolt loosening identification method by calculating the exposed length of the screw using vision-based deep learning and geometric imaging theory, and the results showed that the average measurement error was limited to within 0.61 mm. However, the relationship between the loosening angle and the exposed length of the screw is still unclear. Pan et al.^32,33^ proposed a bolt looseness detection method based on 3D vision. The bolts in the 3D model are located and extracted based on the CNN convolutional neural network and point cloud processing algorithm, and the height of the screw is used to determine whether the screw is loose.

The above studies mainly focused on the quantitative nut rotation and the exposed height of the screw. However, less research has focused on identifying loosening of anti-loosening bolts. Therefore, in this paper, a new anti-loosening bolt loosening detection method is proposed.

## Proposed method

### Bolts with regular hexagonal caps

**Figure 2:**
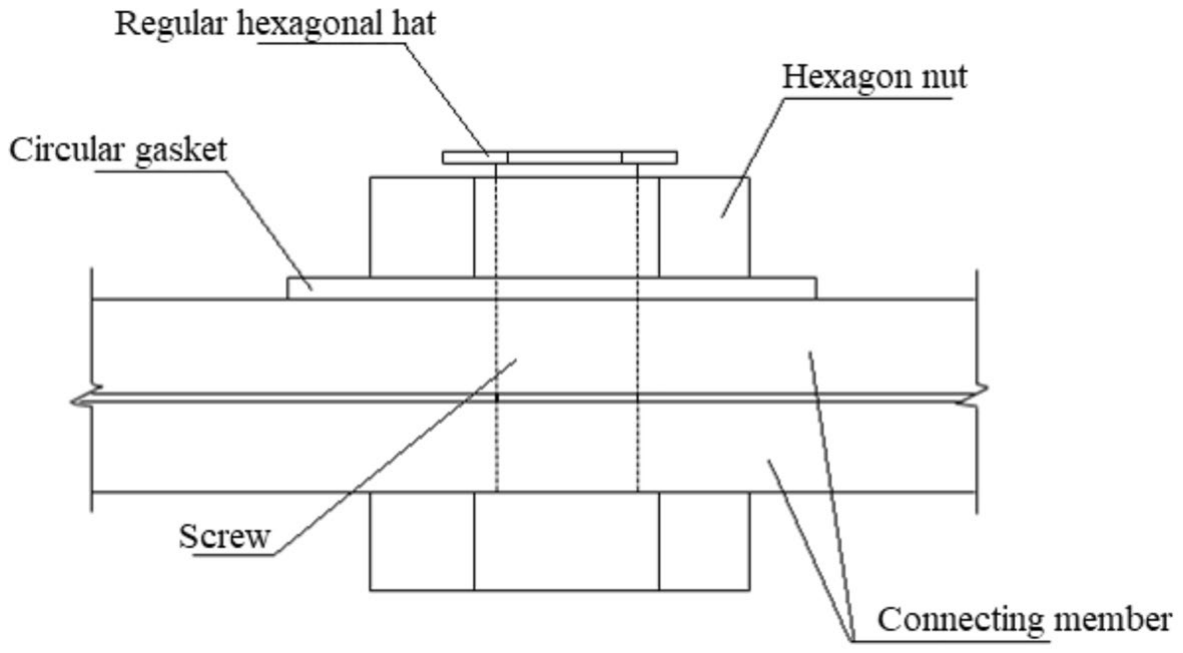
Vertical view of the constructed bolt.

**Figure 3:**
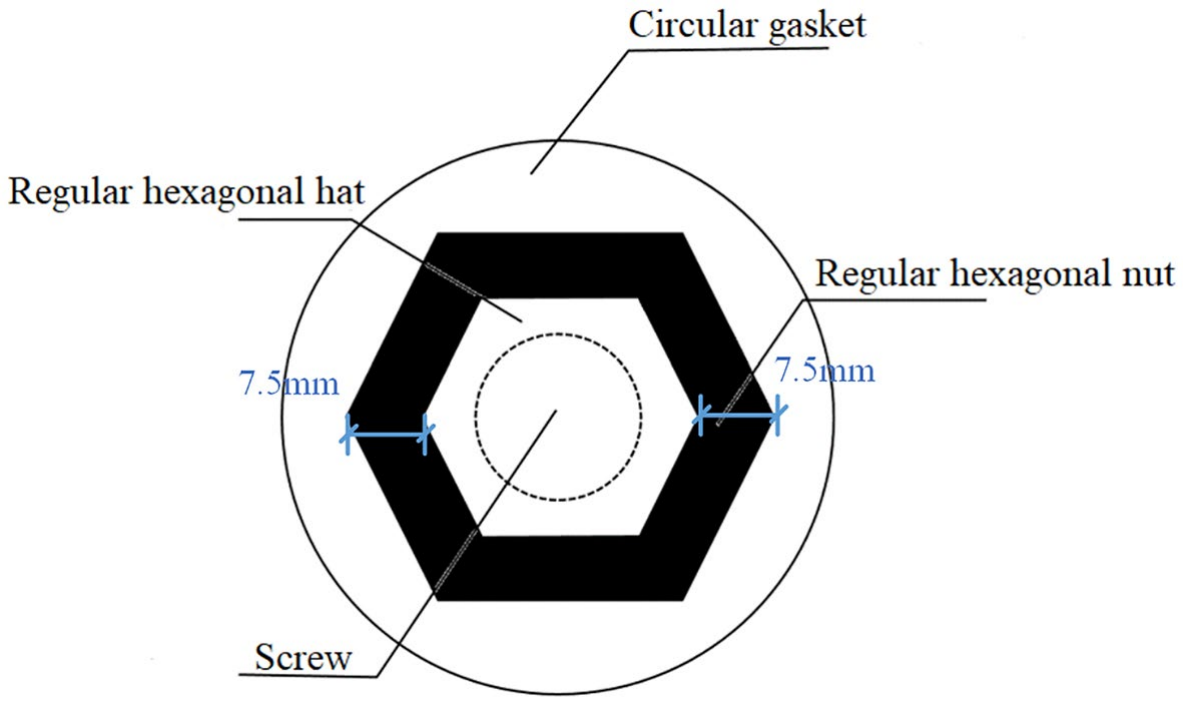
Top view of the constructed bolt.

The vertical and top views of the proposed anti-loosening bolt construction in this paper are shown in Figs. 2 and 3, respectively. An additional regular hexagonal cap was installed at the end of the screw. The distance between the screw cap and nut hexagons was 7.5 mm, as shown in Fig. 2. Except for the regular hexagonal cap, the other bolts are the same as the normal anti-loosening bolt, including the screw, nut, circular washer, and connected components. The regular hexagonal cap is white, and the nut is black, which can maximize the gray gradient difference between the screw cap and nut edge^34^ and improve the stability of edge detection.

### The proposed hexagonal border reconstruction algorithm

**Figure 4:**
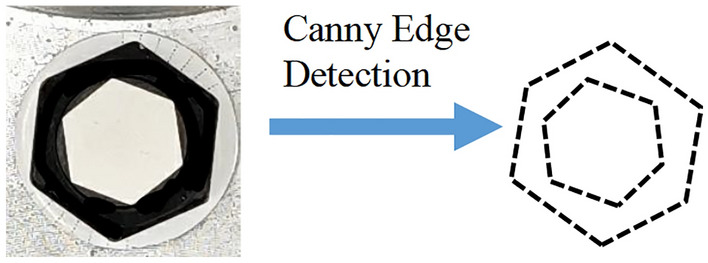
Bolt picture and the hexagonal borders.

When photos of the anti-loosening bolt are obtained using a camera at the top, the hexagonal borders of the screw cap and nut can be determined using the Canny edge detection method, as shown in Fig. 4. However, the relative rotation angle between the screw cap and nut is the desired quantity. Therefore, a new hexagonal border reconstruction algorithm must be proposed to automatically distinguish the hexagonal borders of the screw cap and nut.

**Figure 5:**
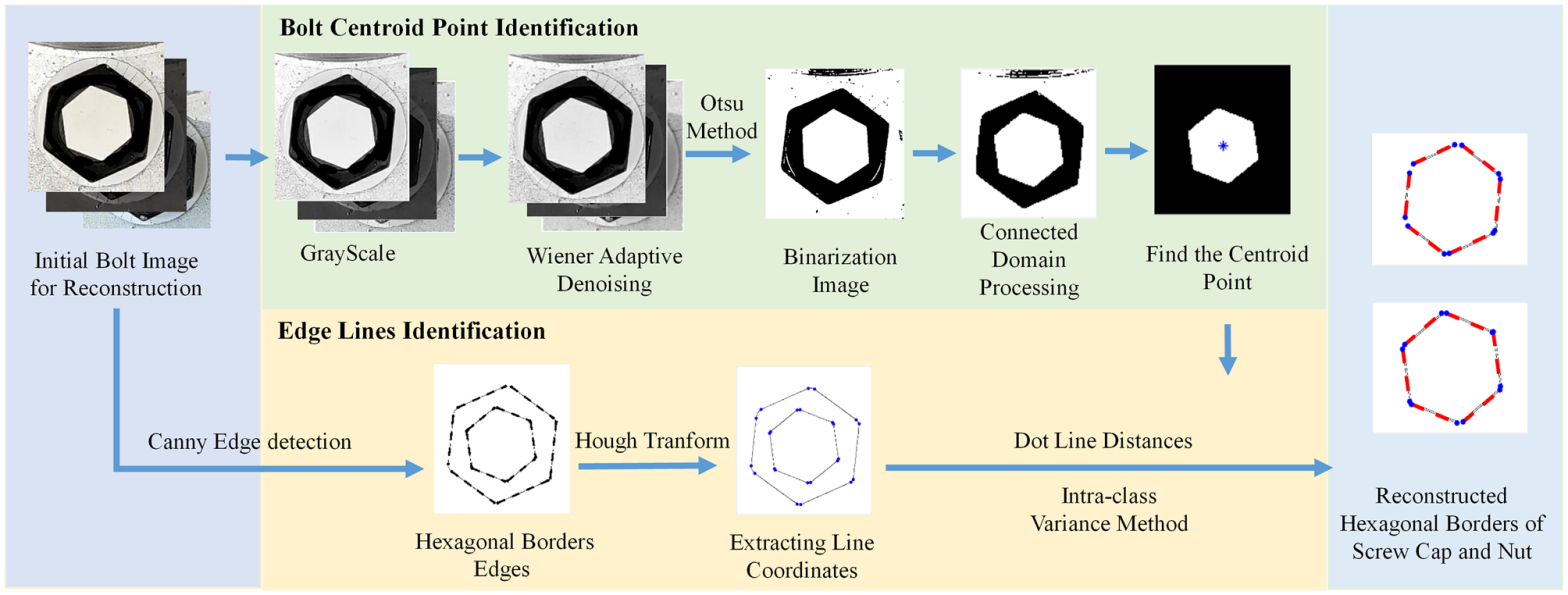
Process of the proposed hexagonal border reconstruction algorithm.

The proposed hexagonal border reconstruction algorithm is shown in Fig. 5. First, the initial image is transformed into grayscale, denoised by a Wiener adaptive filter^35^, and binarized by the Otsu method^36,37^. Second, connected domain processing and morphological operations are used, and the morphological operations usually include erosion, expansion, deletion of interfering binary image areas, calculation of binary image centroid coordinates, etc. Then, the interference areas are deleted, and the centroid coordinates of the regular hexagonal cap can be obtained. Third, the edge lines of the nut and regular hexagonal cap can be segmented. The Canny algorithm^8^ and Hough algorithm^9^ are used to detect edge lines on the initial bolt image, and the endpoint coordinates of the straight edge lines can be extracted. Then, the distances between all endpoint coordinates and the identified centroid point of the regular hexagonal cap can be calculated, and the intra-class variance method^38–40^ is used to adaptively calculate the optimal segmentation distance, which can be used to segment the edge lines of the nut and the regular hexagonal cap. Finally, the hexagonal borders of the nut and regular hexagonal cap can be reconstructed using individual edge lines. Since the Otsu threshold segmentation method can maximize the difference between the front and rear backgrounds and is not affected by image brightness, the Canny algorithm is not sensitive to image brightness. Therefore, the program in this section is not affected by image brightness and has high stability.

### Relative rotation angle of the screw cap and nut hexagons

**Figure 6:**
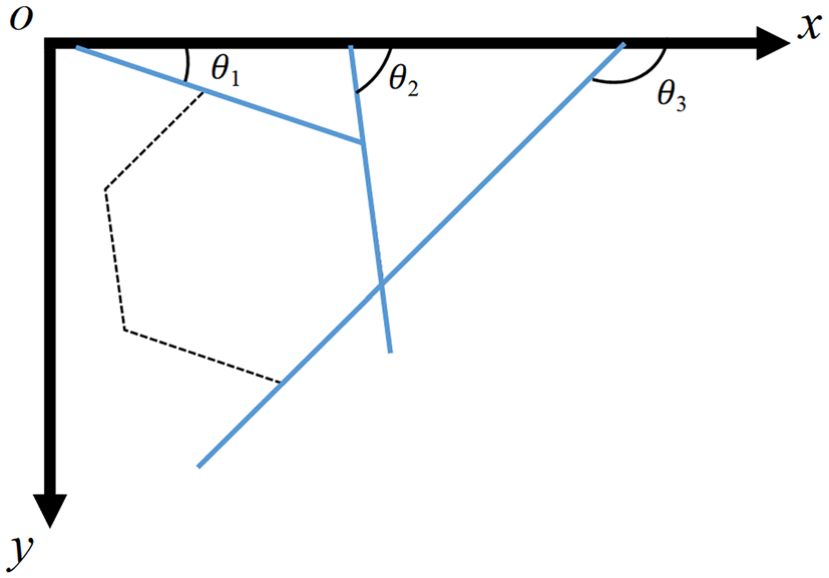
Three angles of a regular hexagonal border.

After reconstructing the hexagonal border of the screw cap or nut, the rotation angle of the screw cap or nut hexagon can be determined. As shown in Fig. 6, a regular hexagonal border has six edges parallel to each other, so there are only three angles on the x-o-y coordinate system, namely, *θ*_1_, *θ*_2_, and *θ*_3_. *θ*_1_, *θ*_2_, and *θ*_3_ have the following angular relationships:

1 $$ \theta_{{2}} = \theta_{{1}} + {6}0^{ \circ } \quad \theta_{{3}} = \theta_{{2}} + {6}0^{ \circ } = \theta_{{1}} + {12}0^{ \circ } . $$

Therefore, when the hexagonal border starts to rotate, the angles *θ*_1_, *θ*_2_ and *θ*_3_ will undergo changes. Thus, based on the relationships among these three angles, the rotation angle of the hexagonal border can be calculated using the angles *θ*_1_, *θ*_2_ and *θ*_3_ as follows:

2 $$ \theta = \frac{{\mathop \sum \nolimits_{j = 1}^{n} rem\left( {\theta_{j} /60} \right)}}{n} $$

where *θ*_*j*_ is the angle between the *j*_th_ edge line and the *x-axis*, *n* is the number of identified sidelines, and *rem*(*.*) is the operator used to determine the remainder after division.

Based on Eqs. (1) and (2), the rotation angle of the hexagonal border of the screw cap can be calculated and defined as *θ*_*inner*_. Similarly, the rotation angle of the hexagonal border of the nut can be calculated and defined as *θ*_*out*_. Then, the relative rotation angle of the screw cap and nut hexagons can be defined as

3 $$ \hat{\theta }{ = }\theta_{inner} - \theta_{out} . $$

### The proposed anti-loosening bolt looseness diagnosis

When the bolt is not loose, the relative rotation angle of the hexagonal borders of the screw cap and nut can be determined and defined as $\hat{\theta }_{u}$. Meanwhile, when the bolt is loose, the relative rotation angle of the hexagonal borders of the screw cap and nut can be determined and defined as $\hat{\theta }_{d}$. Then, the bolt looseness angle of the anti-loosening bolt can be expressed as:

4 $$ \theta^{ * } { = }\hat{\theta }_{d} - \hat{\theta }_{u} . $$

**Figure 7:**
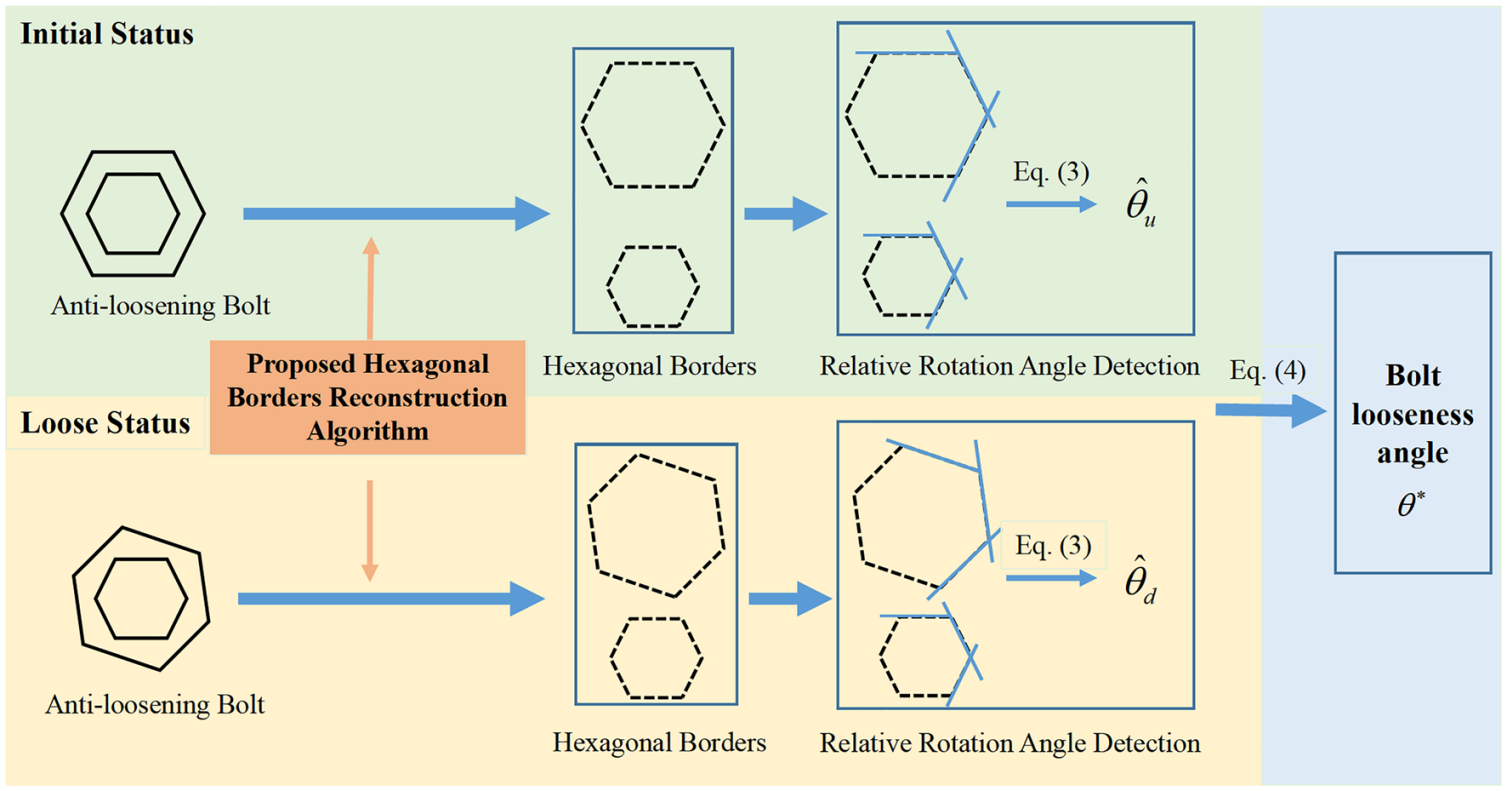
The process of the proposed anti-loosening bolt looseness diagnosis method.

In other words, the proposed method in this paper involves the installation of a regular hexagonal screw cap, the reconstruction of hexagonal borders, the identification of the relative rotation angle of the screw cap and nut, and the comparison of the relative rotation angles under the initial status and loose status. The process of the proposed anti-loosening bolt looseness diagnosis method is shown in Fig. 7.

## Experimental

### Experimental overview

**Figure 8:**
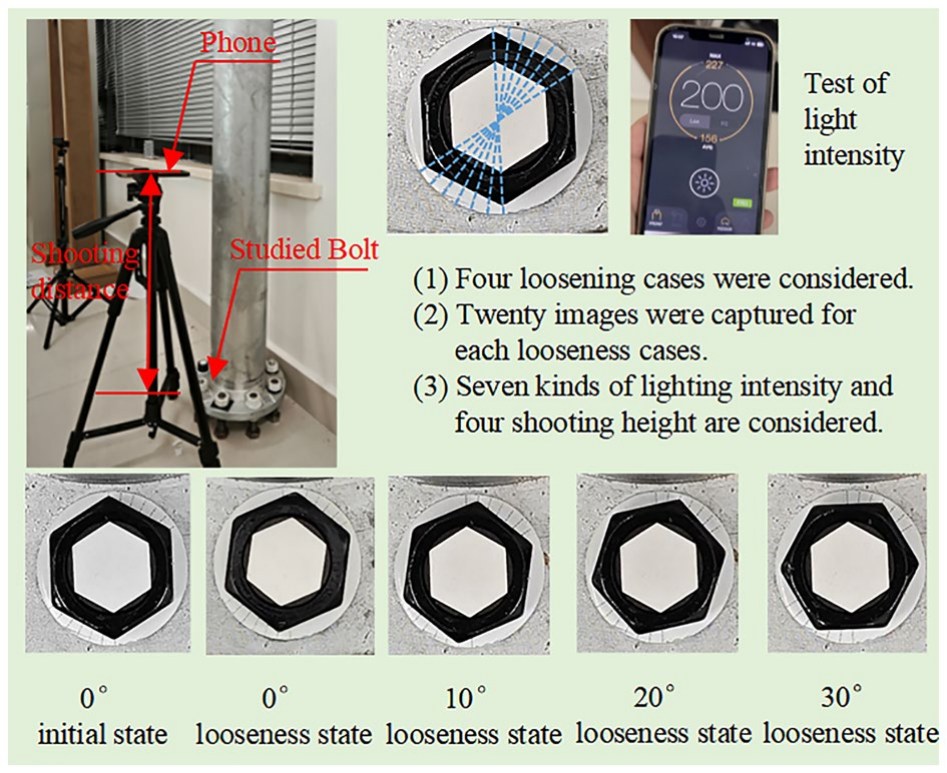
Experimental overview.

To verify the feasibility of the proposed method, a laboratory experiment is implemented. A prototype flange node of the transmission tower was used, as shown in Fig. 8. The hexagonal cap and the screw were connected by an adhesive. One of the bolts was selected for study, and images were captured using an iPhone 12 in the laboratory.

In the experiment, one initial bolt status is considered. Four bolt looseness cases were considered, i.e., the relative rotation angles of the screw cap and nut hexagons were 0°, 10°, 20°, and 30°. For the 0° bolt looseness case, the screw and nut rotate synchronously, and the location of the screw cap and nut is not the same as that for the 0° initial status, as shown in Fig. 7. For the 10°, 20°, and 30° bolt looseness cases, only the nut rotation and looseness angles are simulated by using the change in the relative rotation angle between the screw cap and nut, as shown in Fig. 7. To set the angles accurately, seven adjacent 10-degree scale lines were drawn on the gasket, as shown in Fig. 7.

The influences of lighting intensity and shooting distance were also studied. Seven lighting intensities, four shooting heights and twelve shooting perspective angles are considered and are discussed in the “Analysis of influencing factors” section.

### The validation experiment of the proposed method

**Figure 9:**
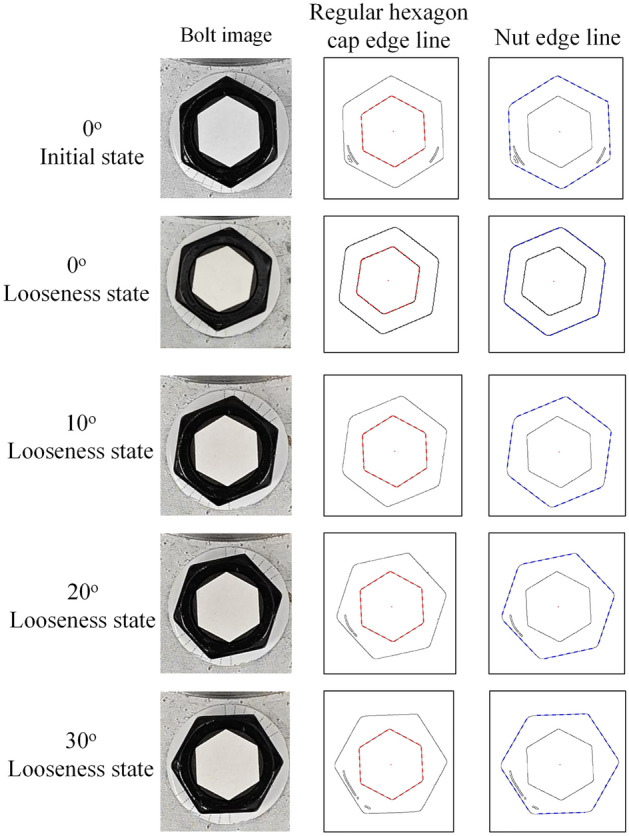
Experimental edge line segmentation results.

Images obtained under a lighting intensity of 200 lx and a shooting height of 50 cm were used to verify the proposed method. Under the initial status, one image was captured to calculate the quantity $\hat{\theta }_{u}$. Under each looseness case, twenty images were captured to study the stability of the identification results. Twenty images under each looseness case were processed, and twenty relative rotation angles $\hat{\theta }_{d}$ between the screw cap and nut were obtained. Examples of edge line segmentation results under different bolt states are shown in Fig. 9.

**Figure 10:**
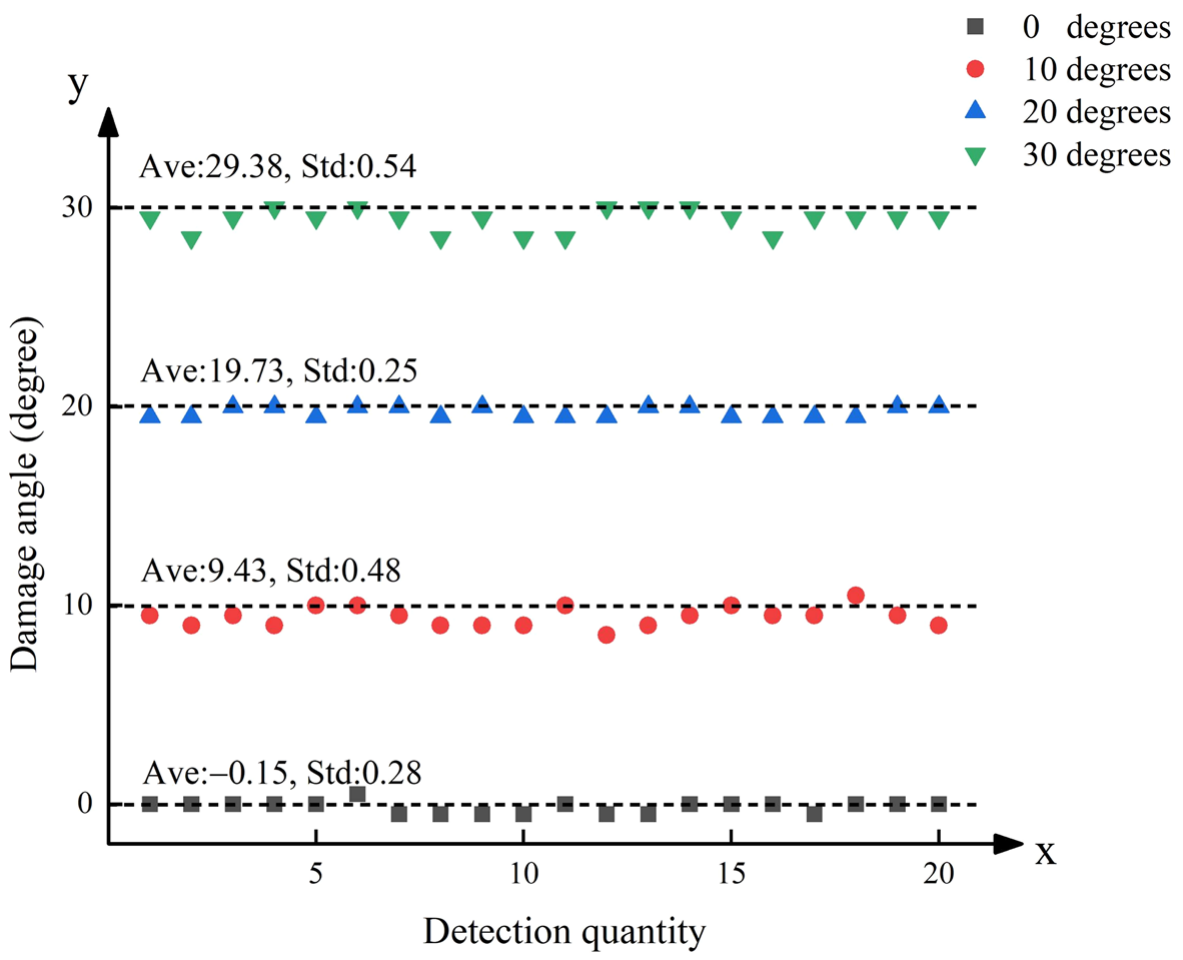
Test results for the 20 bolt looseness cases.

Then, based on Eq. (4), twenty looseness angles θ* can be calculated for each looseness case, as shown in Fig. 10. Ave represents the average value of 20 test results per group. Std represents the standard deviation of 20 test results per group. The results in Fig. 10 show that the mean value deviations are small, with a maximum of only 0.62° under the 30° looseness case. The standard deviations do not exceed 0.54° under the 30° looseness case. The proposed method has good effectiveness and feasibility and high accuracy.

### Comparison of the proposed method and existing methods

**Figure 11:**
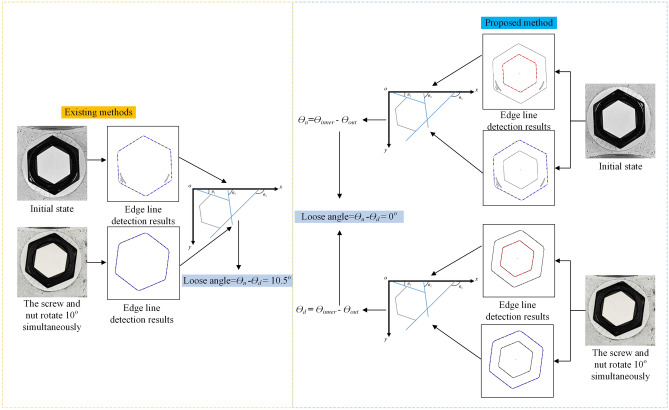
Comparison of the proposed method and existing methods.

One of the images under the 0° initial state and 0° looseness state are used to compare the proposed method and existing bolt looseness quantitative identification methods^13^. To make the calculation process more intuitive, the calculation results obtained using the two methods are shown in Fig. 11. Based on the existing method, the results show that the bolt is already loose and that the rotation angle is 10.5°. In contrast, based on the proposed method, the results show that there is no loose bolt. The method proposed in this article can effectively avoid the impact of the synchronous rotation of the nut and screw on bolt looseness detection.

### Analysis of the influencing factors

To study the influence of camera height, light intensity and shooting perspective angle, bolt looseness detection tests under different test conditions were implemented. Seven lighting intensities, four shooting heights and twelve shooting perspective angles are considered.

### Effect test and result analysis of different shooting heights

**Figure 12:**
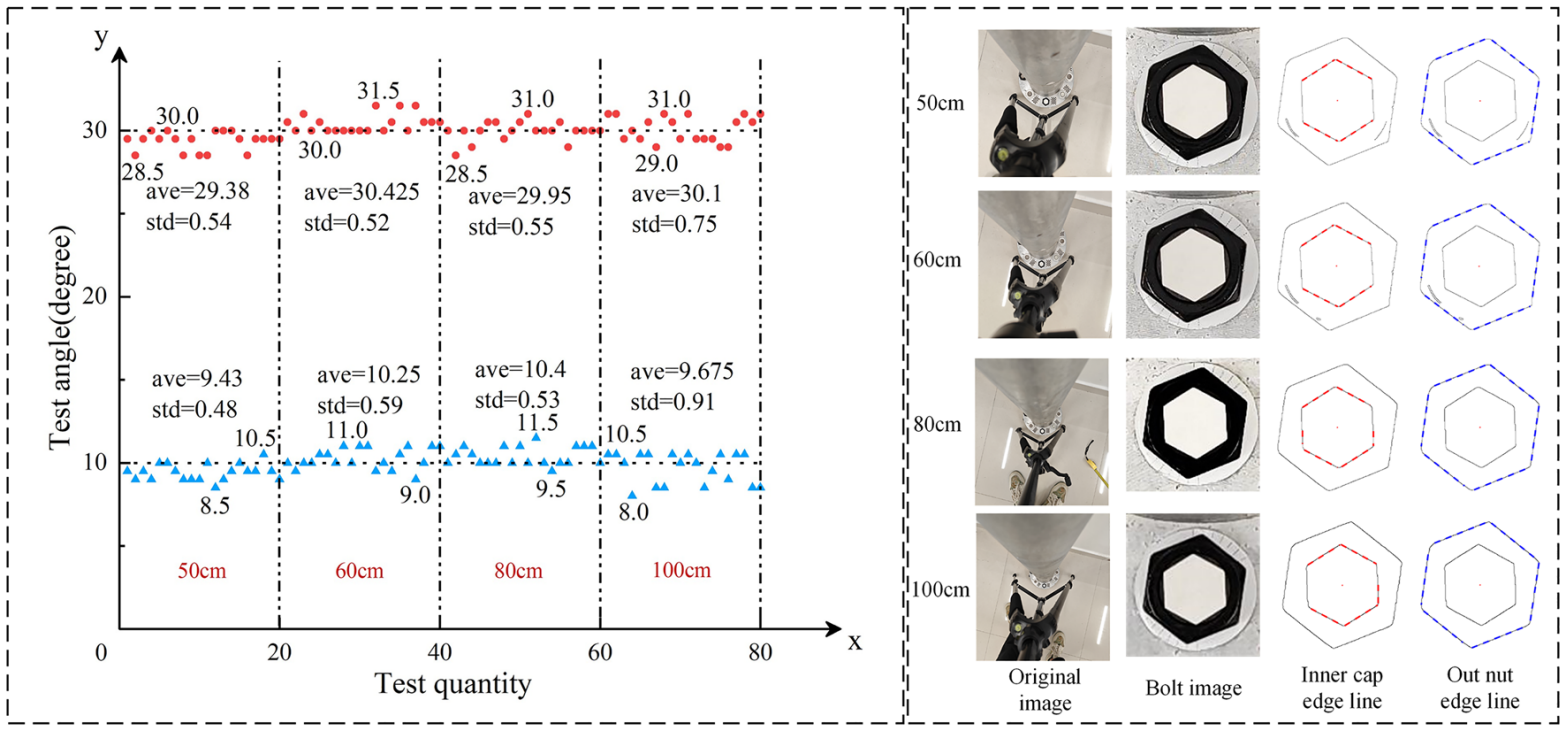
Results for different shooting heights.

Four shooting heights are considered, i.e., 50 cm, 60 cm, 80 cm and 100 cm. The minimum shooting height is limited by the integrity of the bolt images and is set to 50 cm. The looseness angles are set to 10° and 30°. The bolt images with different shooting heights under 10° looseness state are shown in Fig. 12. The indoor lighting intensity was measured as 200 lx. The image acquisition and calculation of the looseness angles θ* are consistent with those in the previous section.

Based on the proposed method, the looseness angles under different shooting heights can be calculated, as shown in Fig. 12. The results show that the impact of shooting height is very limited. Generally, for all the shooting heights, the maximum mean deviation is 0.62° under the 30° looseness case, and the maximum standard deviation is 0.91° under the 10° looseness case, which are still within an acceptable range. However, it is not recommended to shoot bolts at a high height. The reason is that the lower the camera height is, the more details the camera captures, the better the edge identification, and the greater the stability of the bolt looseness detection.

### Effect test and result analysis of different lighting intensities

**Figure 13:**
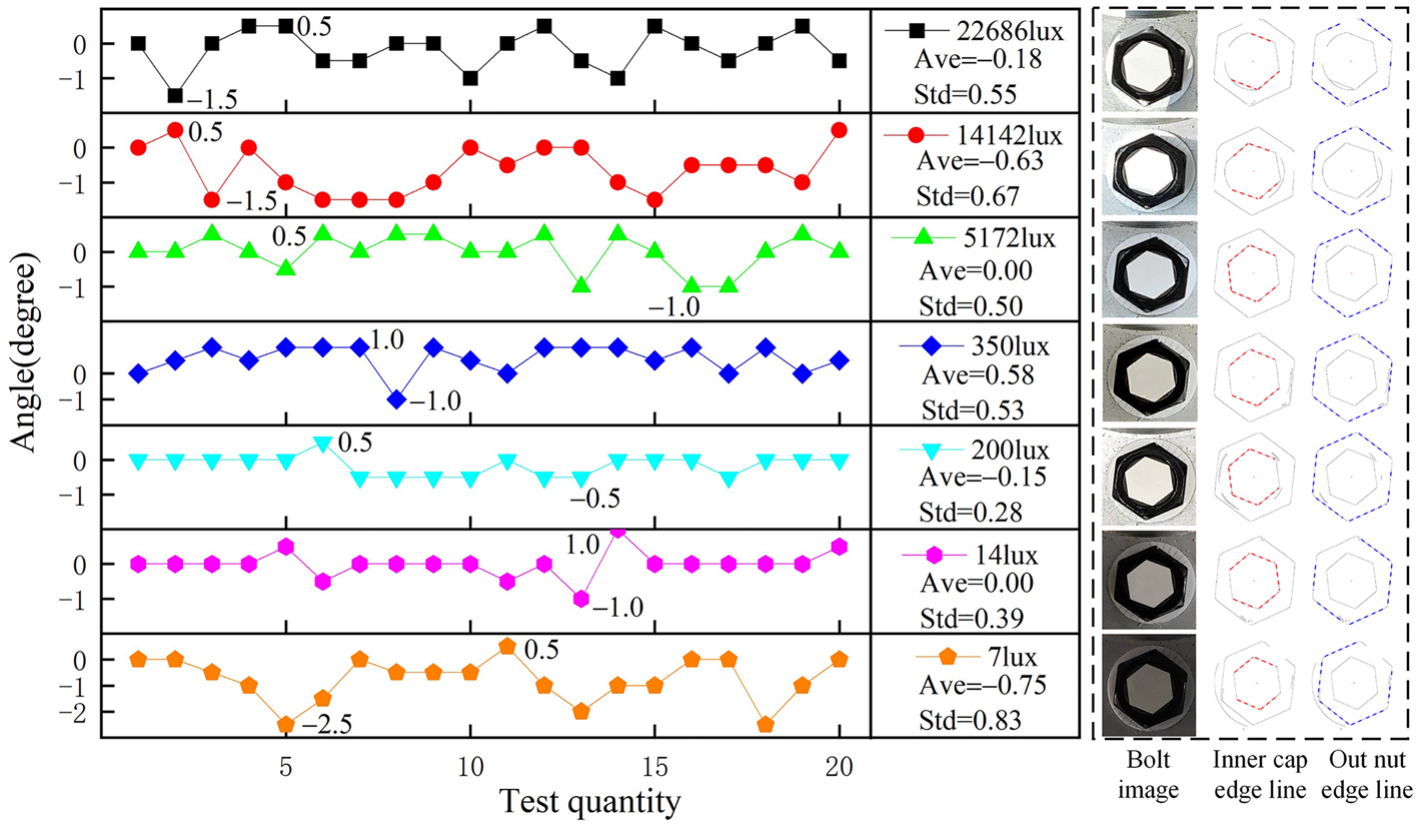
Results under different lighting intensities.

Seven light intensities are considered, i.e., 22,686 lx, 14,142 lx, 5172 lx, 350 lx, 200 lx, 14 lx and 7 lx. The lighting intensities correspond to outdoor strong sunshine weather, outdoor sunshine weather, outdoor bright cloudy weather, outdoor cloudy weather, indoor weather, outdoor evening, and outdoor night, respectively. The shooting height is set as 50 cm. The bolt images with different light intensities under 10° looseness state are shown in Fig. 13. Under each light intensity, 20 bolt images have been obtained. In order to better showcase its fluctuations under different light intensities, the first image under each light intensity is used as the initial state. Therefore, the looseness angle is actually set to 0°. The image acquisition and calculation of the looseness angles θ* are consistent with those in the previous section.

Based on the proposed method, the looseness angles under different lighting intensities can be calculated, as shown in Fig. 13. Figure 13 shows that there is not much difference between the mean value deviations under different light intensities, and the impact of light intensity is very limited. The mean deviation is limited to between -0.75° and 0.58°, which is within an acceptable range. However, the standard deviations under strong or dark light intensities are generally greater than those under normal light intensities, which means that there is greater fluctuation in the identified results under strong or dark light intensities. Under a strong lighting environment, the luminescence on the bolt interferes with imaging, reducing the stability of the proposed method. In a dark lighting environment, the number of noise points in the image will increase. Hence, it is not recommended to shoot images under extreme lighting environments, such as strong or dark lighting environments. In a strong lighting environment, the luminescence on the bolt interferes with imaging. In a dark lighting environment, the number of noise points in the image will increase.

### Effect test and result analysis of different shooting perspective angles

**Figure 14:**
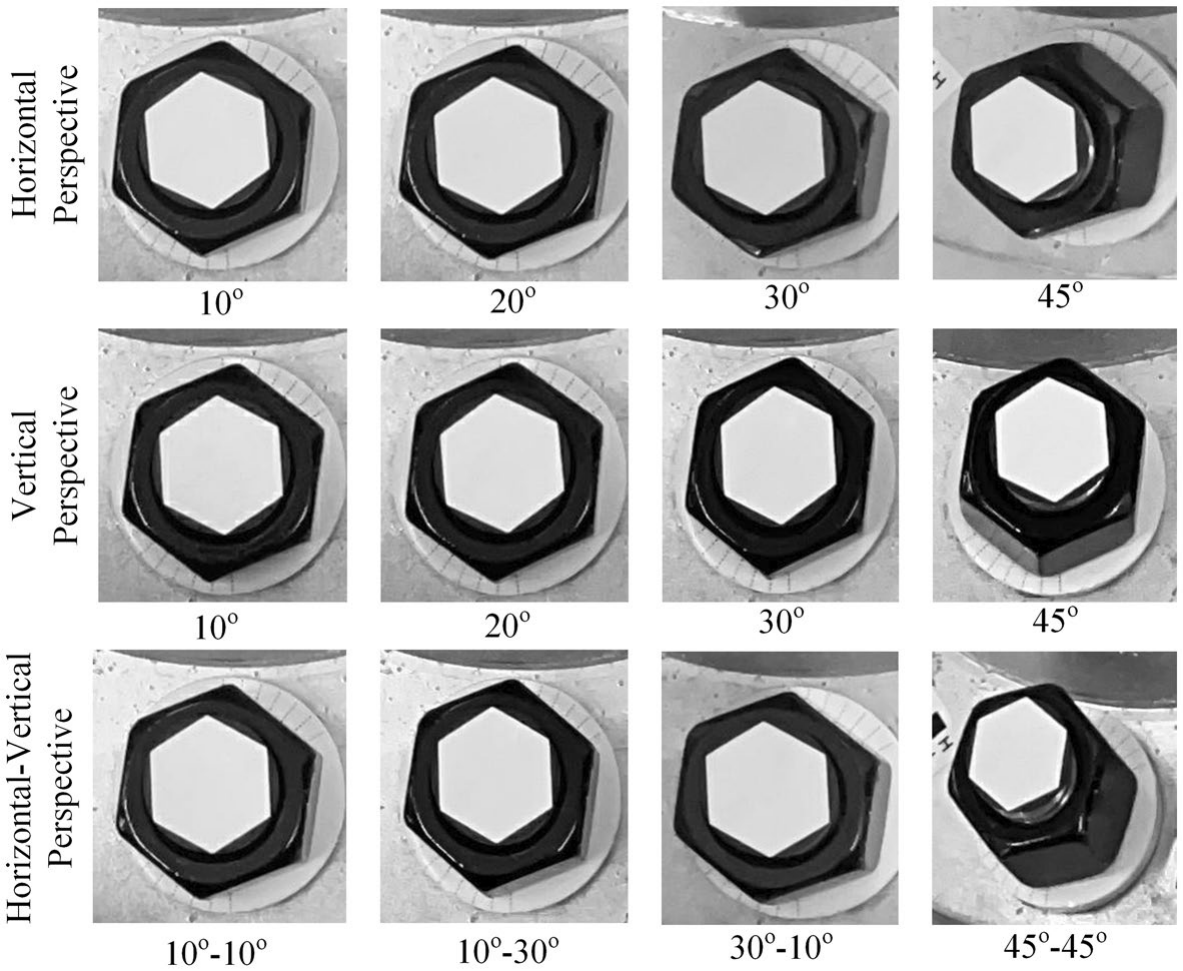
Schematic diagram of bolt images from different perspective angles under a 10° looseness state.

The considered shooting perspective angles include horizontal perspective, vertical perspective, and horizontal-vertical perspective angles. The horizontal and vertical perspective angles are set to 10°, 20°, 30° and 45°. The horizontal-vertical perspective angles are set to 10°–10°, 10°–30°, 30°–10° and 45°–45°. The looseness angles are set to 0°, 10° and 30°. The camera shooting height was set to 50 cm within an indoor environment. A series of 20 images were captured under each perspective angle and each looseness state. Figure 14 displays some bolt images with various perspective angles under a 10° looseness state.

**Figure 15:**
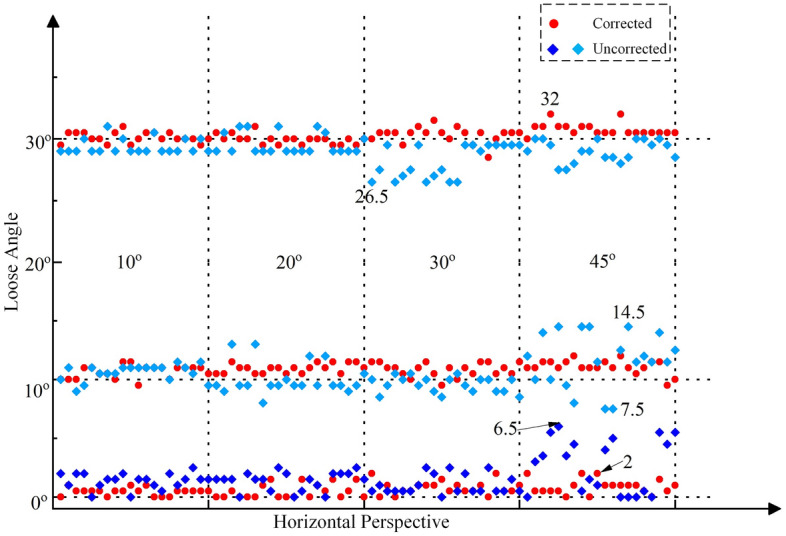
Calculated bolt looseness angles from a horizontal perspective under different looseness states.

**Figure 16:**
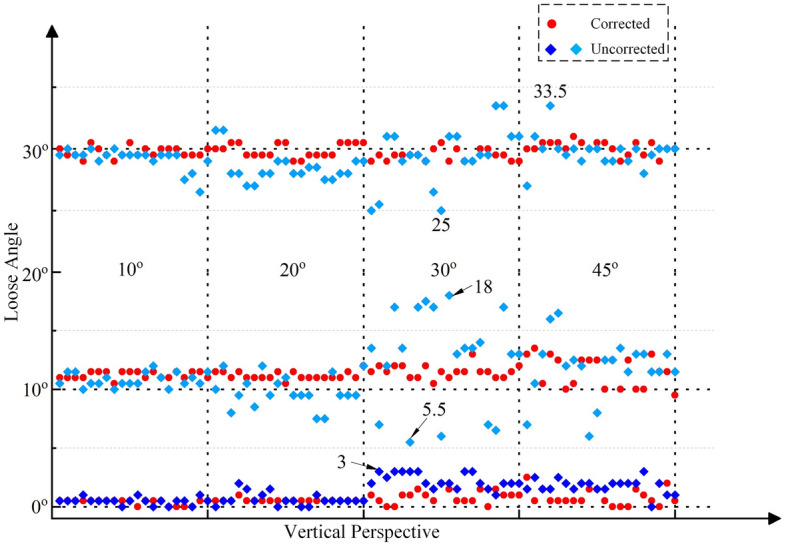
Calculated bolt looseness angle from the vertical perspective under the different looseness states.

**Figure 17:**
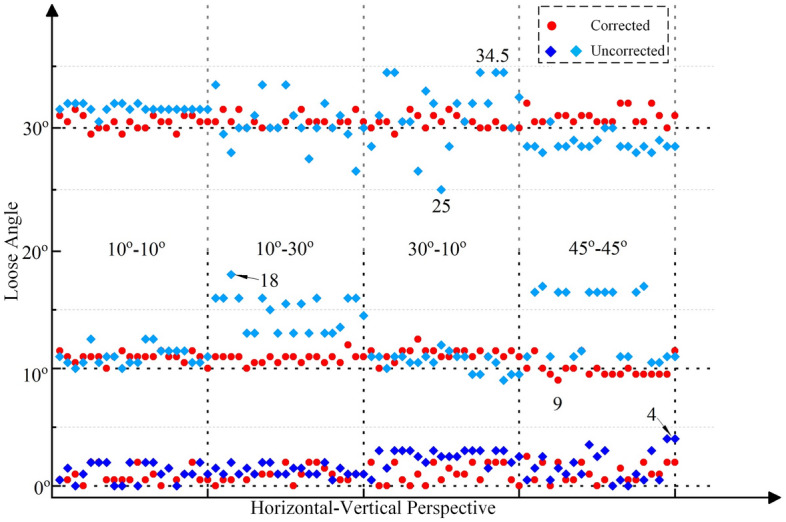
Calculated bolt looseness angle from the horizontal-vertical perspective under the different looseness states.

**Figure 18:**
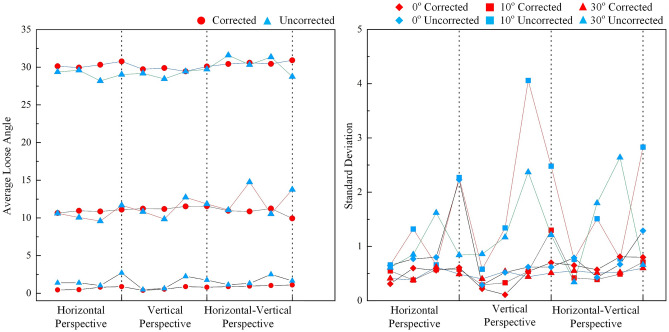
Average and standard deviation values of the anti-loosening bolt loosening diagnostic results for different perspective angles.

Then, the collected images first undergo distortion correction, and the distortion correction method based on the homography matrix is used^41^. Finally, based on the corrected images and the proposed method in this paper, the looseness angles can be calculated, as shown in Figs. 15, 16 and 17. The average and standard deviation values of the results are shown in Fig. 18. The results show the following:

1. The perspective angle has a significant impact on the bolt looseness angle. The identification results using bolt images without perspective angle correction fluctuate more significantly. The maximum fluctuation is from the 0° looseness state and 45° horizontal perspective angle, where the maximum identification error reaches 6.5°. Furthermore, based on the standard deviation results, when the perspective angle is greater than 20°, the fluctuations in the identification results increase when using uncorrected bolt images.
2. The distortion correction method based on the homography matrix is effective. Using the corrected images, the fluctuation of the results is small. The maximum fluctuation is from the 0° looseness state and 45°–45° bidirectional perspective angle, where the maximum identification average value error is 1.1°.
3. In summary, the perspective angle has a significant impact on loosening angle identification. However, the distortion correction method based on the homography matrix is effective, and the impact of the perspective angle on the identification of loosening of the anti-loosening bolt can be effectively reduced.

## Conclusion

This paper proposes a novel anti-loosening bolt looseness diagnosis method using a vision-based technique. A regular hexagonal cap was installed on the screw. Then, a new hexagonal border reconstruction algorithm is proposed to automatically distinguish the hexagonal borders of the screw cap and nut. Finally, a novel anti-loosening bolt looseness diagnosis method is established by using the relative rotation angle of the regular hexagonal borders of the screw cap and nut under initial status and loose status. A prototype flange node is used to verify the proposed method. Four bolt looseness cases, four shooting heights, seven lighting intensities and twelve shooting perspective angles were considered in the experiment. The results show that the proposed method can effectively detect the looseness angle of anti-loosening bolts. Meanwhile, it should be noted that only the standard hexagonal bolts are concerned in this paper and the applicability of the method to other bolt feature needs further discussion.

## Data Availability

All data generated or analysed during this study are included in this published article.
